# *Saccharomyces cerevisiae* DNA Ligase IV Supports Imprecise End Joining Independently of Its Catalytic Activity

**DOI:** 10.1371/journal.pgen.1003599

**Published:** 2013-06-27

**Authors:** Kishore K. Chiruvella, Zhuobin Liang, Shanda R. Birkeland, Venkatesha Basrur, Thomas E. Wilson

**Affiliations:** 1 Department of Pathology, University of Michigan, Ann Arbor, Michigan, United States of America; 2 Department of Molecular, Cellular, and Developmental Biology, University of Michigan Ann Arbor, Michigan, United States of America; 3 Department of Human Genetics, University of Michigan, Ann Arbor, Michigan, United States of America; Duke University, United States of America

## Abstract

DNA ligase IV (Dnl4 in budding yeast) is a specialized ligase used in non-homologous end joining (NHEJ) of DNA double-strand breaks (DSBs). Although point and truncation mutations arise in the human ligase IV syndrome, the roles of Dnl4 in DSB repair have mainly been examined using gene deletions. Here, Dnl4 catalytic point mutants were generated that were severely defective in auto-adenylation *in vitro* and NHEJ activity *in vivo*, despite being hyper-recruited to DSBs and supporting wild-type levels of Lif1 interaction and assembly of a Ku- and Lif1-containing complex at DSBs. Interestingly, residual levels of especially imprecise NHEJ were markedly higher in a deletion-based assay with Dnl4 catalytic mutants than with a gene deletion strain, suggesting a role of DSB-bound Dnl4 in supporting a mode of NHEJ catalyzed by a different ligase. Similarly, next generation sequencing of repair joints in a distinct single-DSB assay showed that *dnl4*-K466A mutation conferred a significantly different imprecise joining profile than wild-type Dnl4 and that such repair was rarely observed in the absence of Dnl4. Enrichment of DNA ligase I (Cdc9 in yeast) at DSBs was observed in wild-type as well as *dnl4* point mutant strains, with both Dnl4 and Cdc9 disappearing from DSBs upon 5′ resection that was unimpeded by the presence of catalytically inactive Dnl4. These findings indicate that Dnl4 can promote mutagenic end joining independently of its catalytic activity, likely by a mechanism that involves Cdc9.

## Author Summary

Chromosomal rearrangements are common driver mutations in human genetic disease and cancer. The junctions observed at rearrangements typically show only a few base pairs in common between the partners, suggesting that they were formed by the end-to-end joining process, nonhomologous end joining (NHEJ). However, there is uncertainty about the mechanisms that actually create mutated junctions. DNA ligase IV catalyzes restorative double-strand break (DSB) joining in the canonical NHEJ pathway, but increasing evidence suggests that distinct NHEJ pathways that use DNA ligases I and/or III might be more important for mutations. We used yeast to study the *in vivo* consequence of having DNA ligase IV that was catalytically inactive but that nonetheless accumulated at DSBs normally. We detected mutated junctions in some assays that required DNA ligase IV protein but not its catalytic activity. This pattern suggests that DNA ligase I creates many mutated junctions when DNA ligase IV is present and that this can become a predominant mode of repair when DNA ligase IV activity is inefficient. Our yeast ligase IV mutations have properties similar to those observed in the human ligase IV syndrome, underscoring the relevance of these observations.

## Introduction

DNA double-strand breaks (DSBs) are potentially catastrophic chromosomal lesions. Accordingly, many proteins are recruited to DSBs for repair by non-homologous end joining (NHEJ) or homologous recombination (HR). NHEJ, a pathway conserved from yeast to humans, repairs DSBs by processing and directly ligating the DNA ends, often with little or no nucleotide loss [1], [2]. In *Saccharomyces cerevisiae*, the core components of the canonical NHEJ (c-NHEJ) machinery are the Ku (Yku70–Yku80), MRX (Mre11–Rad50–Xrs2) and DNA ligase IV (Dnl4–Lif1–Nej1) complexes [3]. The final step in c-NHEJ is DNA ligation, carried out by the DNA ligase IV catalytic subunit (Dnl4 in yeast, Lig4 in humans) in complex with its scaffolding protein XRCC4 (Lif1 in yeast) and supported by the XRCC4-like factor XLF (Nej1 in yeast) [1], [3], [4].

Dnl4/Lig4 is an ATP-dependent DNA ligase that functions exclusively in NHEJ [1], [3], [4]. The ATP-dependent DNA ligation mechanism has three distinct catalytic steps [4]. In step 1, the enzyme displaces pyrophosphate from ATP leading to covalent auto-adenylation of the ligase on its active site lysine (K282 in Dnl4), a reaction that can be reversed by incubating adenylated enzyme with excess pyrophosphate. This intermediate is long-lived such that most cellular ligase molecules are adenylated. In step 2, the activated AMP is transferred to a DNA 5′ phosphate via a 5′-5′ phosphoanhydride bond, which is finally cleaved in step 3 by the attack of an adjacent 3′ hydroxyl leading to DNA ligation and release of AMP. DNA ligases undergo profound conformational changes upon ATP and DNA binding [5], [6]. While the above properties are common to all DNA ligases, Dnl4/Lig4 has other unique but poorly understood properties presumably related to its role in DSB ligation, including an ability to ligate incompatible ends [7] and a slow rate of auto-adenylation [8].

There are many potential consequences of failed c-NHEJ ligation. Most importantly, 5′ resection of persistent DSB ends generates 3′-terminated single-stranded DNA tails that are essential for HR [9], [10]. Loss of c-NHEJ proteins in yeast, including Dnl4, has been reported to lead to increased rates of 5′ resection and HR [11], [12], suggesting that either the bound c-NHEJ protein complex and/or c-NHEJ ligation is in competition with HR. Additionally, because error-free HR is not always possible, DSB-derived deletions and other mutations catalyzed by alternative NHEJ (alt-NHEJ) pathways [13]–[16] or catastrophic chromosome loss and cell death can result from c-NHEJ ligation failure. The importance of these negative consequences is underscored by the DNA ligase IV deficiency syndrome, in which human patients with impaired Lig4 function display microcephaly, dysmorphology, developmental delay, bone marrow and immune deficiency, radiosensitivity and malignancy [17], [18].

The balance of the above outcomes might depend substantially on the reason for c-NHEJ ligation failure. However, most genetic studies of Dnl4/Lig4 function have been performed with gene deletion mutants, while most human mutations and polymorphisms are point changes [19]. For these reasons, we have performed mutational analysis of Dnl4 in *S. cerevisiae*, where an extensive set of outcomes can be assessed. We describe a distinct and mutagenic NHEJ repair behavior of a series of mutations in the ligase catalytic domain as compared to complete loss of the Dnl4 protein. Despite being severely catalytically defective, these mutants, along with the c-NHEJ complex including Ku and Lif1, hyper-accumulated at induced DSBs and supported imprecise NHEJ in some assays. Cdc9 likely represents the alternative ligase used in the presence of dysfunctional Dnl4, as evidenced by its appearance there in a time frame consistent with NHEJ catalysis. Finally, both Dnl4 mutants and Cdc9 were efficiently removed from DSBs and presented no impediment to 5′ resection, suggesting an active switch from NHEJ to HR.

## Results

### Dnl4 Catalytic Mutants Are Stable and Bind Lif1 Normally

**Figure 1:**
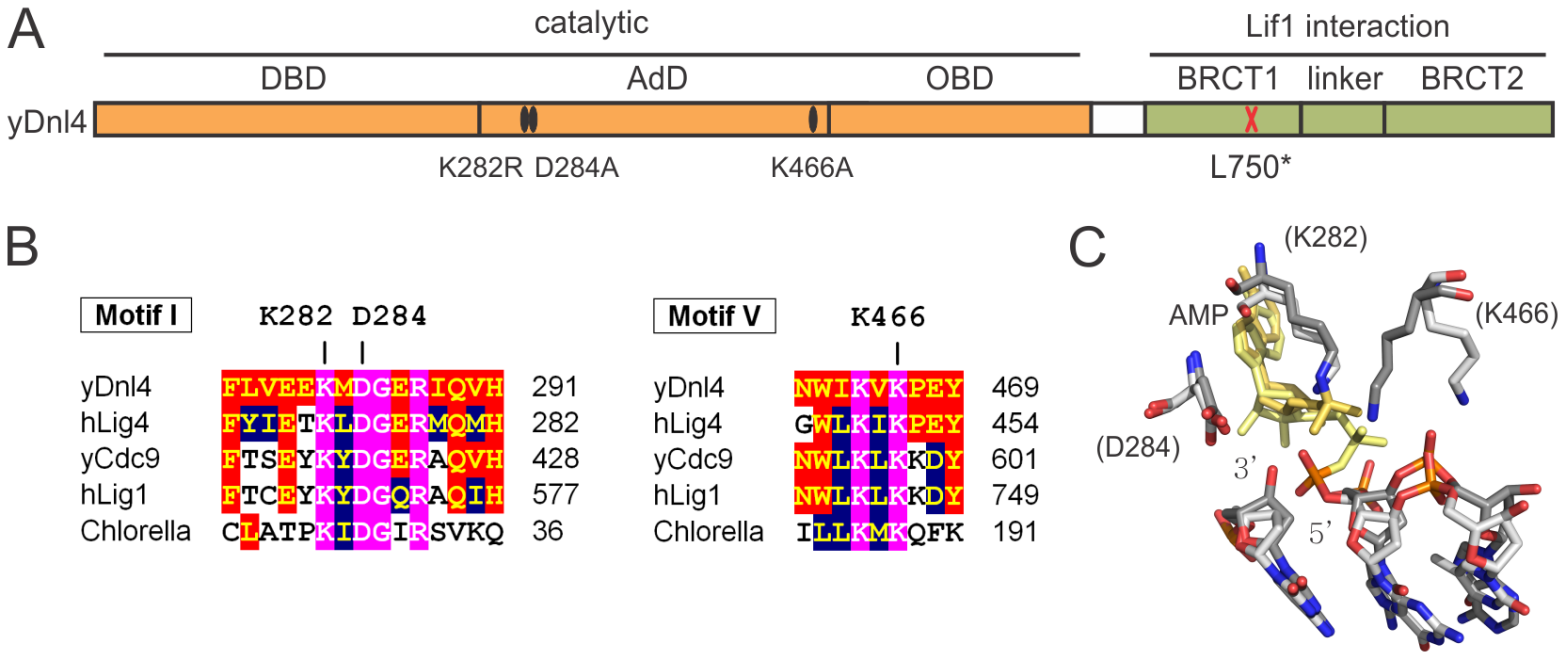
Dnl4 mutations under study. (**A**) Location of mutations made in this study relative to the functional domains of *S. cerevisiae* Dnl4 (yDnl4). DBD, DNA binding domain; AdD, adenylation domain; OBD, oligonucleotide binding domain; BRCT, BRCA1 C-terminal repeat; black oval, point mutation; red cross, stop codon. (**B**) Multiple sequence alignments surrounding conserved mutated yDnl4 positions. hLig4, human DNA ligase IV; yCdc9, *S. cerevisiae* DNA ligase I; hLig1, human DNA ligase I; Chlorella, chlorella virus DNA ligase. Magenta, identical among all proteins; red, identical to yDnl4; blue, conserved relative to yDnl4. (**C**) DNA ligase catalytic active site showing a structural alignment of hLig1 bound to a 5′-adenylated DNA nick (PDB 1X9N [5], shaded more lightly) and adenylated Chlorella virus ligase bound to a nick (PDB 2Q2T [22], shaded more darkly). Shown are the AMP (yellow), the substrate DNA strand with labeled 3′ and 5′ nick termini, and the universally conserved residues under study, labeled as the homologous positions in yDnl4. Protein and DNA are shaded by element.

**Figure 2:**
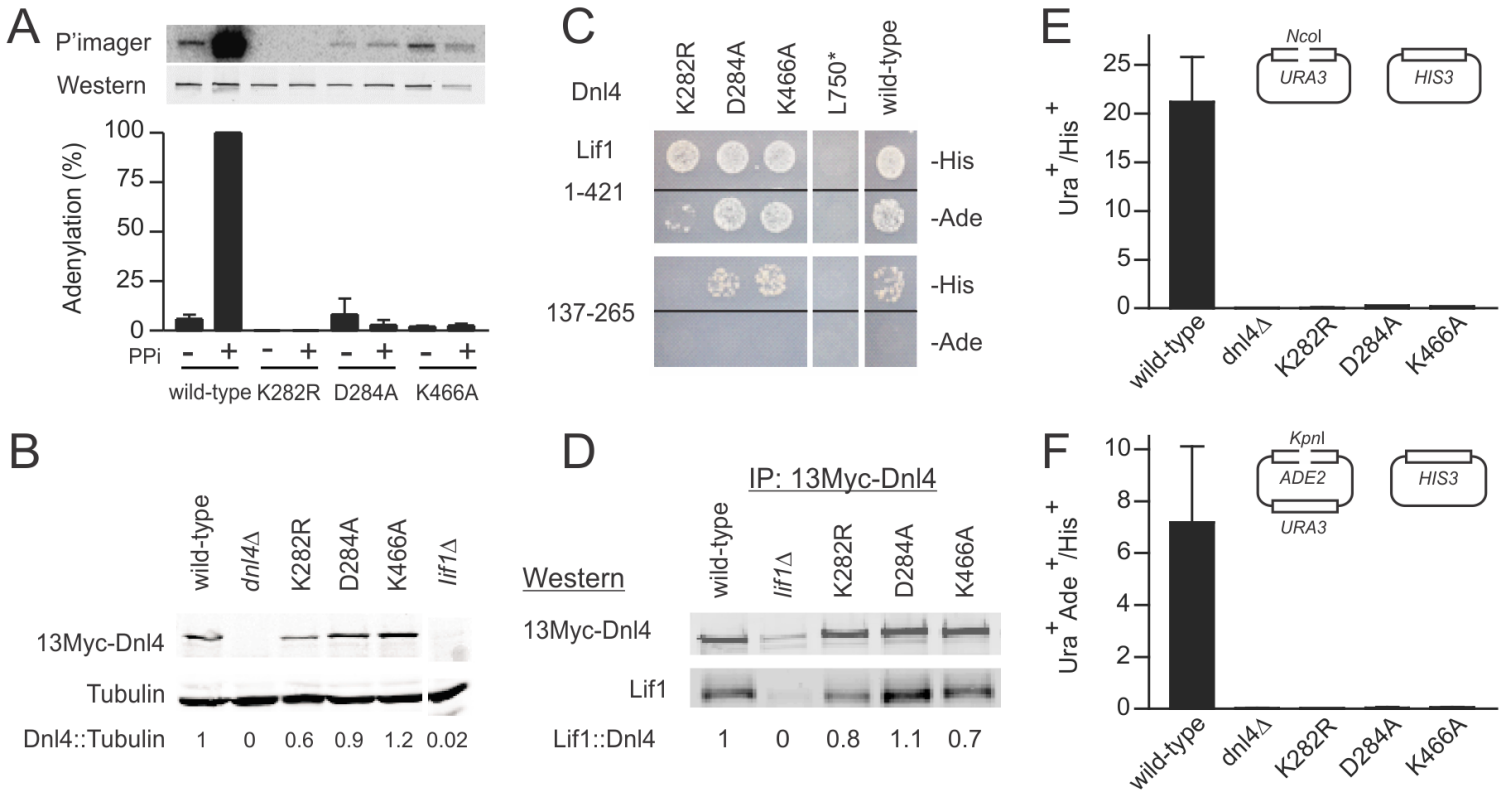
Dnl4 catalytic mutations severely impair auto-adenylation and c-NHEJ efficiency. (**A**) Immunoprecipitated 13Myc-Dnl4 was incubated with α-^32^P ATP to form a radiolabeled enzyme-adenylate complex, with or without sodium pyrophosphate (PPi) pre-treatment to remove pre-existent covalently bound AMP. Phosphorimager (top panel) and immunoblot (bottom panel) exposures of an example blot are shown, as well as normalized results over three independent experiments (bottom panel). (**B**) Immunoblot of whole cell extracts from yeast expressing 13Myc-tagged Dnl4 using anti-Myc and anti-tubulin antibodies (Santa Cruz Biotechnology and Thermo Scientific, respectively). The normalized Dnl4 to tubulin signal ratio is shown. (**C**) Dnl4-Lif1 yeast two-hybrid analysis. Haploid strains expressing the full-length Dnl4 constructs (baits) were mated with strains expressing Lif1 1–421 or 137–265 (preys). The Dnl4-Lif1 interaction was scored by spotting to plates lacking either histidine (-His) or adenine (-Ade). (**D**) 13Myc-Dnl4 protein was immunoprecipitated from yeast in the same manner as (A) and immunoblotted for co-immunoprecipitated native Lif1 (antibody a kind gift Alan Tomkinson). Numbers are the Lif1/Dnl4 ratio normalized to wild-type Dnl4, after correcting for the background band co-migrating with Lif1. (**E**) and (**F**) Plasmid recircularization assay with 5′ and 3′overhangs, respectively. Results are the mean ± standard deviation of at least two independent experiments.

We introduced mutations into the *DNL4* chromosomal locus to explore the DSB repair behavior of catalytically defective Dnl4 (Figure 1A). Mutations in ligase Motif 1 (K282R, D284A) and Motif V (K466A) affect universally conserved residues predicted to be involved in ligation catalysis based on comparisons to structure-function studies of viral DNA ligases [20]–[22] and the crystal structure of human DNA ligase I [5] (Figure 1B,C). These residues were chosen on the hypothesis that they might differentially affect the three steps of ligation. K282R alters the lysine predicted to form the covalent AMP adduct and should be an obligatory step 1 mutant, a behavior validated by the complete inability of immunoprecipitated Dnl4-K282R to become adenylated *in vitro* (Figure 2A). By analogy to viral ligase mutants [20], [21] it was possible that D284A and K466A might be step 2 and step 3 mutants, respectively. However, each of these mutant proteins showed severely reduced *in vitro* adenylation following pre-incubation with excess pyrophosphate to remove pre-existent AMP adducts (Figure 2A). Because adenylation and pyrophosphorolysis are reverse reactions, these data indicate that Dnl4-D284A and Dnl4-K466A are also severely defective in ligation step 1.

We next examined the dependence of ligase stability and the Dnl4-Lif1 interaction on catalytic function. Western blots of whole cell extracts demonstrated that Dnl4-K282R was expressed at moderately reduced levels relative to wild-type Dnl4 (Figure 2B), suggesting a partial instability of this mutant form. In contrast, Dnl4-D284A and Dnl4-K466A were expressed normally (Figure 2B). These expression results mirrored the apparent strength of the Dnl4-Lif1 interaction as revealed by two-hybrid analysis (Figure 2C), but in fact all three catalytic mutants showed a good ability to co-immunoprecipitate Lif1 (Figure 2D). We interpret that the reduced Dnl4-K282R-Lif1 two-hybrid signal is most likely due to reduced Dnl4 expression rather than a defect in Lif1 binding, and thus that Dnl4 catalytic function is not required for Lif1 binding.

For comparison in repair studies below, we also created truncation mutant L750*, which lacks the core of the Lif1 interaction region [23] (Figure 1A). As expected, Dnl4-L750* was unable to bind Lif1 (Figure 2C).

### Dnl4 Catalytic Mutants Are Strongly Defective in C-NHEJ

To determine the c-NHEJ phenotype of the catalytic Dnl4 mutants, corresponding strains were transformed by linearized plasmids bearing 5′ (*Nco*I) or 3′ (*Kpn*I) overhanging DSBs, which is known to depend on Dnl4-mediated simple-religation c-NHEJ [24]–[26]. Consistent with their severe adenylation defect (Figure 2A), all three mutants were impaired in plasmid repair to nearly the same degree as a *dnl4Δ* mutant (Figure 2E,F), confirming the expected finding that catalysis by Dnl4 is required for c-NHEJ.

### Catalytically Defective Dnl4 Binds To, and Is Removed From, Chromosomal DSBs

**Figure 3:**
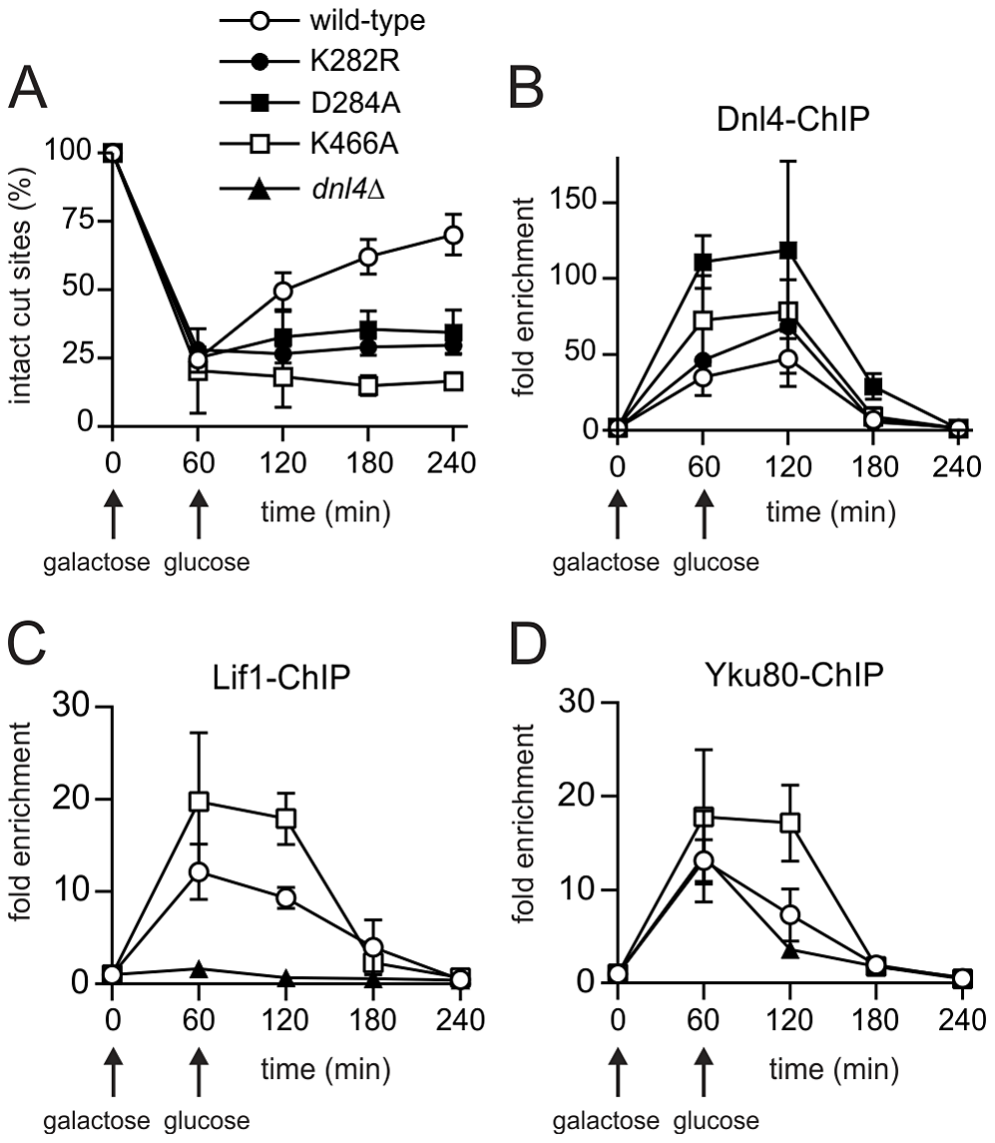
Extensive recruitment of catalytically inactive Dnl4 and associated c-NHEJ factors to a chromosomal DSB. Yeast strains bearing the indicated Dnl4 mutations and the *GAL1*-cs allele were grown in galactose medium for 60 min to induce HO expression. Cells were then transferred to glucose to allow repair by NHEJ. (**A**) The fraction of intact *GAL1*-cs HO cut sites showing the extent of DSB formation and repair over time, determined by flanking PCR. (**B**) The corresponding enrichment of 13Myc-tagged Dnl4 at the *GAL1*-cs DSB relative to the *ACT1* control locus as determined by ChIP from the same samples as (A). (**C**) Enrichment of 13Myc-tagged Lif1 and (**D**) 13Myc-tagged Yku80 at the *GAL1*-cs DSB. Results are the mean ± standard deviation of at least two independent experiments.

To determine whether catalytically defective Dnl4 mutants are recruited to DSBs *in vivo*, we employed a previously described system, *GAL1*-cs, in which a single DSB is introduced into the chromosomal *GAL1* promoter by transient induction of HO endonuclease [27]. In this system, the accumulation of 13Myc-tagged Dnl4 at the DSB is measured by chromatin immunoprecipitation (ChIP) and any associated NHEJ repair is monitored by a parallel quantitative PCR assay. Unlike wild-type Dnl4, which showed an increase in intact HO cut sites from 24% at one hour to 72% at four hours after DSB induction, Dnl4-K282R, -D284A and -K466A were all unable to repair the cleaved allele (Figure 3A), consistent with results above. ChIP demonstrated that the NHEJ defect of the catalytic mutants was not a result of failed recruitment to the DSB. The three mutant proteins each accumulated at the DSB at one and two hours and in fact achieved even higher levels of binding than wild-type (Figure 3B), presumably because DSBs in these strains were not being repaired.

Further ChIP analysis was used to explore whether an intact c-NHEJ complex, or just Dnl4, was accumulating at DSBs. Similar to Dnl4, Lif1 was hyper-recruited to DSBs at one and two hours in *dnl4*-K466A as compared to the wild-type strain, in marked contrast to the greatly impaired accumulation of Lif1 observed in our system and others in a *dnl4*Δ strain (Figure 3C) [11]. Yku80 showed a similarly enhanced ChIP signal in the *dnl4*-K466A mutant, while signal was slightly reduced at two hours in the *dnl4*Δstrain in a manner that likely reflects the reported stabilization of Ku by the ligase complex (Figure 3D) [11]. Thus, we conclude that typical, stable, but non-productive c-NHEJ complexes are assembled at DSBs in strains bearing catalytically defective Dnl4.

ChIP readouts also provided key information regarding the dissociation of Dnl4. We previously observed that Dnl4 and other c-NHEJ proteins begin rapidly disappearing from DSBs after two-hours in an MRX-dependent fashion [27], [28]. Figure 3B–D demonstrate that this disappearance is independent of successful c-NHEJ because non-productive c-NHEJ complexes were removed from DSBs with the same time course as when Dnl4 had successfully catalyzed DSB repair in many cells.

### NHEJ, but Not Bound Dnl4, Restricts DSB Resection

We previously showed that disappearance of c-NHEJ proteins from DSBs correlates with the onset of 5′ resection in preparation for HR [27]. Others have suggested that the presence of DNA ligase IV and the c-NHEJ complex at a DSB is inhibitory to resection [11], [12]. However, the gene deletion mutations used in such studies both prevent productive c-NHEJ and disrupt c-NHEJ assembly. This distinction is important because experiments that rely on continued expression of HO allow for futile cycles in which DSBs are created, repaired and recreated. Observed effects of *dnl4Δ* mutants might result from interruption of this repair cycle rather than direct inhibition of resection by Dnl4. Dnl4 catalytic mutants provided a means of distinguishing these phenomena.

**Figure 4:**
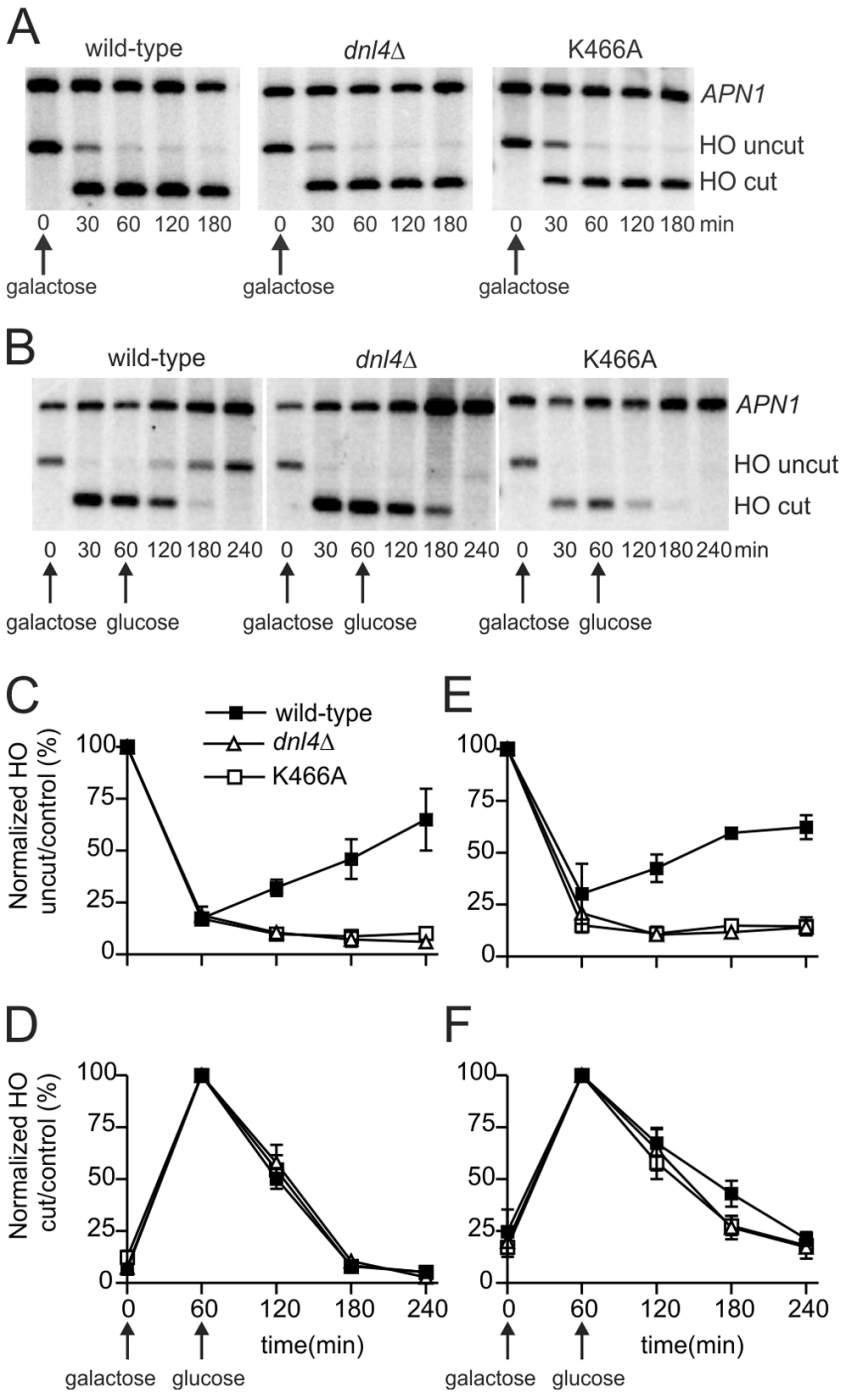
Catalytically inactive Dnl4 protein does not impede DSB resection. DSBs were induced in yeast strains bearing the *ILV1*-cs allele similarly to Figure 3 and monitored by Southern blotting. (**A**) Blot showing that DSB resection is not observed without the addition of glucose at 60 min. (**B**) Example blot showing formation of the HO-cut band and its subsequent disappearance by a combination of NHEJ and DSB resection. (**C**) and (**E**) The ratio of the HO-uncut band to the *APN1* control was normalized to the ratio at time 0 to allow monitoring of DSB formation and repair by NHEJ. (**D**) and (**F**) The ratio of the HO-cut band to the *APN1* control was normalized to the ratio at 60 min when DSB formation was maximal. Disappearance of the HO-cut band at subsequent times results from NHEJ (wild-type strain only) and/or DSB resection. (**C**) and (**D**) show results from asynchronous cells while (**E**) and (**F**) show results from cells arrested in G1 with α-factor. Results are the mean ± standard deviation of five independent experiments.

For monitoring resection we used a previously described system, *ILV1*-cs, in which a single HO DSB is introduced into the chromosomal *ILV1* promoter [27]. Here a one-hour induction period was followed by the addition of glucose to turn off HO expression, so that either NHEJ or 5′ resection might ensue. These two downstream events were monitored and distinguished by Southern blotting. Surprisingly, glucose addition proved to be essential for DSB resection to occur (compare Figures 4A and 4B). We attribute this to the fact that our strains are *gal1* due to fusion of HO to the native *GAL1* promoter and therefore cannot utilize galactose as a carbon source. Consistently, reintroduction of the *GAL1* gene on a plasmid restored resection without the addition of glucose (Figure S1), typical of an extensive yeast literature at various DSB sites. These results demonstrate that the metabolic state of a cell substantially influences resection efficiency.

Figure 4B–D shows *ILV1*-cs results with asynchronous cultures of yeast expressing wild-type Dnl4, no Dnl4 (*dnl4Δ*) or Dnl4-K466A. Once again, substantial NHEJ occurred upon glucose addition to the wild-type strain that was absent with both *dnl4* mutants (Figure 4B,C). This repair contributed to the disappearance of the HO-cut alleles (Figure 4B,D). However, loss of the HO-cut band also reflects competing 5′ resection, and indeed it also disappeared from the *dnl4* mutant strains. The rate of loss of the HO-cut band was equivalent regardless of whether productive c-NHEJ was occurring. This result indicates that resection did occur more robustly in the *dnl4* mutant strains but that this difference was accounted for by the DSBs being repaired by c-NHEJ in wild-type. Moreover, the rate of HO-cut band loss, and thus resection, was identical when comparing *dnl4Δ* and K466A strains (Figure 4D). Similar to previous results [9], [11], [12], G1 arrest inhibited resection, although with *ILV1*-cs system this effect was incomplete (Figure 4E,F). In G1, we did observe a mildly accelerated loss of the HO-cut band in *dnl4* mutant strains as compared to wild-type, similar to prior observations [11]. However, the resection rate was again no different between *dnl4Δ* and K466A (Figure 4F). Similarly, there was no difference in HR-mediated repair when a homologous donor allele was introduced into the various strains (Figure S2). We conclude that the increased rate of resection in *dnl4* mutants can be attributed to the loss of competing NHEJ and is not correlated with the presence of Dnl4 at a DSB.

### Dnl4 Promotes Imprecise NHEJ Independently of Its Catalytic Function

**Figure 5:**
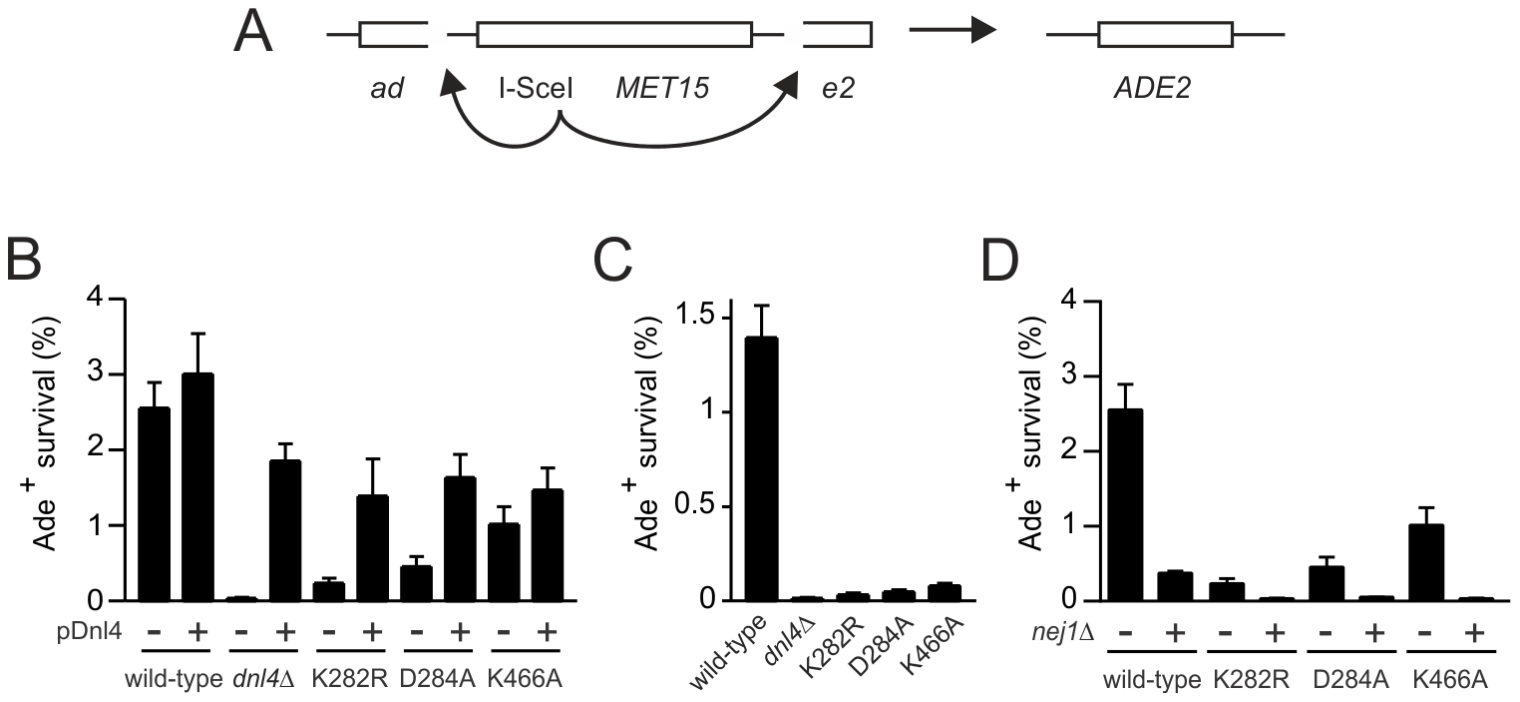
Residual NHEJ in the chromosomal suicide deletion assay with catalytically defective Dnl4. (**A**) Diagram of the suicide deletion chromosomal assay used to determine NHEJ efficiency in panels (B) to (D). (**B**) Cells were pre-grown to stationary phase in synthetic defined medium prior to plating to galactose. When indicated, the chromosomal *dnl4* allele was complemented with a plasmid bearing wild-type *DNL4* (p*DNL4*). (**C**) Cells were pre-grown to log phase in YPA-Glycerol. (**D**) Epistasis analysis of Dnl4 catalytic mutants with *nej1*Δ. Results are the mean ± standard deviation of at least three independent experiments.

We further examined the NHEJ function of the catalytic mutants at two nearby chromosomal DSBs using the well-described suicide deletion assay [26], [29], [30]. Suicide deletion involves galactose-induced formation of two I-*Sce*I DSBs flanking an internal cassette that are joined to recreate the *ADE2* gene (Figure 5A). With this assay, *dnl4*-L750* was as deficient as *dnl4Δ* yeast (Table 1), confirming the known Dnl4 dependence of the assay [26], [30]. In marked contrast, we observed measureable rates of Ade^+^ colony formation with all of the Dnl4 catalytic mutants above that observed with *dnl4Δ* and L750* (Table 1 and Figure 5B). Critically, Ade^+^ colonies can only arise when the split halves of *ADE2* are restored by NHEJ. To verify this, we re-expressed I-*Sce*I in them and observed that, as expected, many repair joints could be re-cleaved by I-*Sce*I and so occurred by simple religation (Table 1). These results indicate that residual end joining capacity exists that depends on Dnl4 protein but not its catalytic activity.

**Table 1:** NHEJ precision in Ade^+^ suicide deletion colonies.

	Joint analysis (%)			Colony Survival (%)			Relative Efficiency (%)		
*DNL4* genotype	I-*Sce*I^+^/total	Precise	imprecise	total Ade^+^	precise	imprecise	total NHEJ	precise	imprecise
**wild-type**	112/120	93	7	2.2	2.0	0.1	100	100	100
***dnl4Δ***	120/120	100	0	0.002	0.002	0	0.1	0.1	0
**K282R**	36/118	31	69	0.2	0.1	0.1	8	3	84
**D284A**	66/117	56	44	0.3	0.2	0.1	15	9	99
**K466A**	190/216	88	12	1.3	1.2	0.2	60	57	109
L750*	120/120	100	0	0.002	0.002	0	0.1	0.1	0

‘I-*Sce*I^+^/total’ indicates the fraction of Ade^+^ survivors that were capable of being re-cleaved by I-*Sce*I, demonstrating that they had used precise NHEJ. ‘Colony Survival’ is the fraction of Ade^+^ colonies that had survived by total, precise and imprecise NHEJ relative to all cells plated. ‘Relative Efficiency’ is the efficiency of total, precise and imprecise NHEJ for mutant strains relative to wild-type Dnl4.

Sequencing the *ADE2* alleles from a number of I-*Sce*I-resistant Ade^+^ colonies revealed a similar and typical collection of in-frame but imprecise NHEJ junctions, including microhomologies, insertions and deletions, for both wild-type Dnl4 and the catalytic mutants (Figure S3A). Indeed, the absolute frequency of imprecise NHEJ was not substantially different for the catalytic point mutants as compared to wild-type Dnl4, in marked contrast to *dnl4Δ* and L750* yeast that showed a complete loss of imprecise junctions (Table 1). Thus, the joining pathway dependent on the Dnl4 protein, but not its activity, was substantially more mutagenic than that being catalyzed by Dnl4.

Ade^−^ suicide deletion colonies are much rarer than Ade^+^ colonies and cannot be selected for when plating, preventing statistically meaningful frequency comparisons. However, we performed allelic analysis on the Ade^−^colonies we did recover (Table 2), first by scoring for status of the *STE3-MET15* marker contained within the cassette that is lost upon suicide deletion (Figure 5A). We then attempted to amplify the DSB junctions from Ade^−^ Met^−^ colonies to determine whether they corresponded to imprecise NHEJ or large deletions of the *ADE2* locus. Strikingly, *dnl4Δ* colonies never arose due to imprecise NHEJ but instead showed high frequencies of large deletions (Table 2). In contrast, the catalytic mutants showed imprecise NHEJ in a pattern that mirrored the Ade^+^ suicide deletion results (Table 2, Figures S3B). We conclude that imprecise NHEJ in the suicide deletion assay depends on Dnl4, but not its catalytic activity.

**Table 2:** Joint analysis of Ade^−^ suicide deletion colonies.

*DNL4* genotype	Met^−^	PCR^+^	Large deletion (%)	Imprecise NHEJ (%)	Other (%)
**wild-type**	25/183	17/25	4	9	86
***dnl4Δ***	32/141	0/26	23	0	77
**K282R**	37/140	3/24	23	3	74
**D284A**	21/140	5/20	11	4	85
**K466A**	27/190	20/25	3	11	86

Ade^−^ Met^−^ colonies are inferred to have undergone suicide deletion, while Met^+^ colonies survived galactose by other means. Met^−^ colonies that were also positive for PCR flanking the expected DSB junction corresponded to imprecise NHEJ (see also Figure S3B), while PCR-negative colonies were inferred to correspond to large deletions. Percentages are calculated relative to all Ade^−^ colonies analyzed for each strain.

The different result pattern when comparing the suicide deletion (Figure 5) and plasmid recircularization (Figure 2E,F) assays was striking, especially since the I-*Sce*I and *Kpn*I 3′ DSBs are in the same position in *ADE2*. Importantly, our standard suicide deletion protocol [26] entails pre-growth of cells to post-diauxic phase for two days, unlike the log-phase cells used in plasmid recircularization assays. Indeed, the residual suicide deletion repair with Dnl4 catalytic mutants was almost entirely suppressed when the experiment was repeated with log-phase cells (Figure 5C), demonstrating that, like resection, the mutagenic end joining pathway dependent on Dnl4 protein is strongly influenced by the metabolic state of the cell.

Residual NHEJ in the above suicide deletion experiments cannot have been catalyzed by Dnl4-K282R as it cannot be adenylated (see Figure 2A). We further assessed the *in vivo* adenylation state of Dnl4-K466A. Overexpressed Dnl4 proteins were excised from a gel (Figure S4A) and subjected to LC-MS/MS. Importantly, enhanced suicide-deletion NHEJ was still seen with overexpressed Dnl4-K466A (). Twelve adenylated peptides were recovered with wild-type Dnl4 out of 163 K282-containing peptides (Figure S4C). This fraction is smaller than expected and likely reveals instability of the adenylation intermediate during fragmentation. Nevertheless, no adenylated peptides were recovered out of 128 K282-containing peptides from Dnl4-K466A, consistent with the *in vitro* results that it is deficient in ligation step 1.

The above results with catalytically defective Dnl4 were reminiscent of prior observations that yeast lacking the Dnl4 accessory protein Nej1 display substantial residual suicide deletion NHEJ, unlike Ku, Lif1 and MRX mutants that phenocopy the near complete defect seen with *dnl4*Δ [30]. However, the residual NHEJ observed with *nej1*Δ and catalytically defective Dnl4 do not reflect the same phenomenon since *nej1 dnl4* double mutants showed a synergistic decrease in suicide deletion survival (Figure 5D). Thus, Nej1 is required for the residual NHEJ observed with catalytically defective Dnl4. In turn, Dnl4 likely catalyzes the residual NHEJ observed with *nej1*Δ, demonstrating that c-NHEJ is differentially dependent on Nej1 and Lif1 in at least this assay.

A final possibility was that the Dnl4 catalytic mutants might act as dominant negatives if they could preclude the action of competent Dnl4 molecules. To test this, we transformed *dnl4* mutant strains with a *DNL4* plasmid. The same degree of complementation was observed with the three catalytic point mutants as with the *dnl4Δ* strain (Figure 5B), indicating that point mutant and wild-type Dnl4 had equal access to the DSB.

### Dnl4-K466A Confers a Different Imprecise Joining Profile than Wild-Type

We next pursued high-throughput next-generation sequencing of NHEJ occurring at HO-induced DSBs in the *ILV1*-cs assay (i) to determine whether the findings regarding imprecise suicide deletion NHEJ could be confirmed using a single-DSB assay, and (ii) to dramatically improve the capacity with which imprecise joints could be scored to allow a more accurate comparison of the joining profiles of different strains. Here we did not add glucose following initial DSB induction in order to suppress 5′ resection (see Figure 4A) and to allow for continuous (re)cleavage of intact HO cut sites and thereby enrich for imprecise joining. Genomic DNA was collected from wild-type, *dnl4*-K466A, and *dnl4Δ* strains at 0-hour and 24-hour time points and PCR products flanking the HO DSB site were subjected to Illumina HiSeq sequencing.

**Figure 6:**
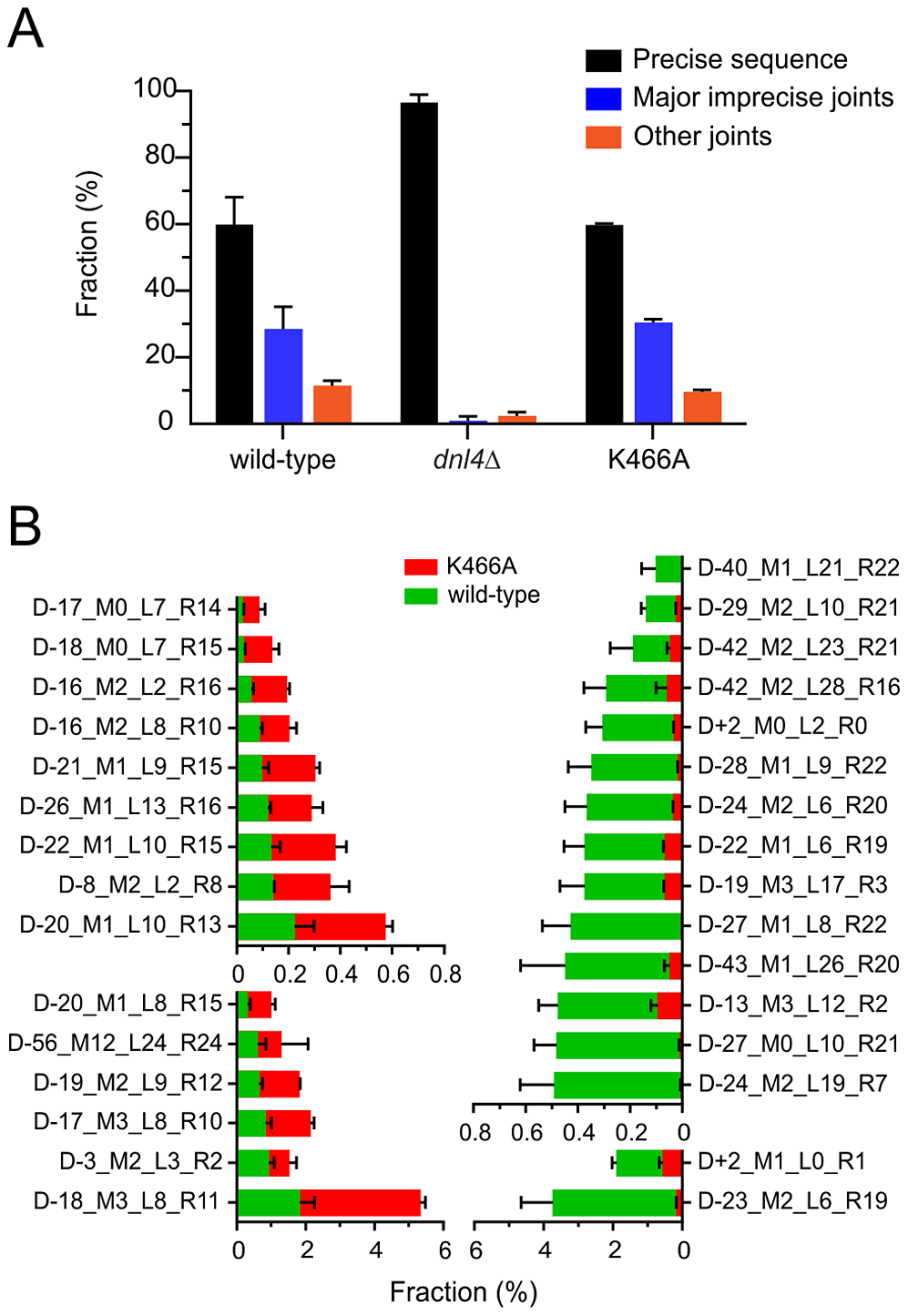
End joining profiles after extensive HO recleavage depend on Dnl4 status. (**A**) Fractions of different *ILV1*-cs joint categories after 24 hours of HO induction, showing preserved imprecise joining with K466A. “Precise sequence” is the same as the input allele prior to DSB formation, “major imprecise joints” had a frequency ≥0.1% in any tested strain, and “other joints” are the remainder. (**B**) Individual major imprecise joints that showed significantly different frequencies between wild-type and K466A. The left half shows joints that had increased frequency in K466A as compared to wild-type. The right half shows joints that had a decreased frequency. Red bars, joint fraction in K466A; green bars, joint fraction in wild-type. See Figure S5 for a description of the joint identifiers and File S1 for their sequences.

As expected, all strains showed ≥98% intact cut sites from among all recovered reads before HO induction (File S1). At 24 hours, wild-type and K466A strains each showed an average across two independent replicates of 40% imprecise joints, consistent with the design of the experiment (Figure 6A). In marked contrast, the *dnl4Δ* strain yielded 97% precise joints and only 3% imprecise joints at 24 hours, a pattern more like the 0-hour time point than the other strains. The recovery of mainly intact cut sites in the most highly NHEJ deficient strain is explained by the presence of uncleaved alleles in dead cells combined with the near absence of *bona fide* repair events in live cells, given that results represent the frequency of sequences recovered from a sample, not their absolute efficiency. The inferred severe impairment of imprecise NHEJ only in the *dnl4Δ* strain closely parallels the findings from the suicide deletion assay.

To compare the imprecise joining profiles of wild-type and Dnl4-K466A, joints were codified to names containing all information needed to uniquely define them (explained in Figure S5). The DESeq program [31] was then used to apply the negative binomial distribution to identify joints for which the frequency was significantly different between the two 24-hour replicates of K466A as compared to wild-type (Figure S6). To help ensure statistical validity, we further restricted our attention to only those joints that had a frequency >0.1% in any tested strain, for which robust counts had thus been obtained (File S1). Although wild-type and K466A strains showed a similar overall frequency for these major imprecise joints (Figure 6A), 15 (25%) and 16 (27%) individual joints showed significantly increased and decreased frequencies, respectively, when comparing K466A to wild-type (Figure 6B). These results establish that loss of Dnl4 catalytic activity confers differences in the quality of imprecise joints that are all nonetheless dependent on the Dnl4 protein.

### Cdc9 Is Recruited to DSBs Coincident with Dnl4

**Figure 7:**
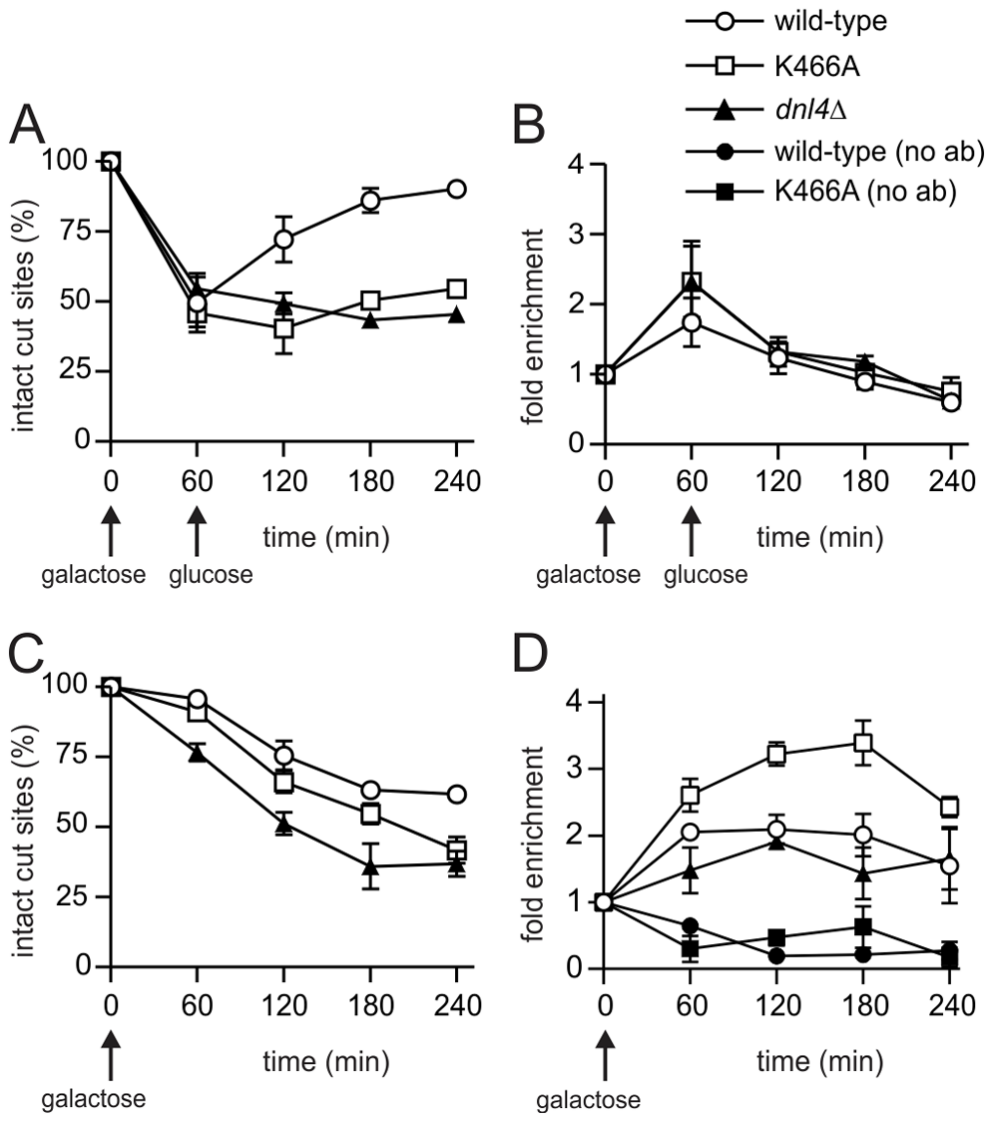
Cdc9 recruitment to a chromosomal DSB during the NHEJ repair phase. Yeast strains bearing 13Myc-tagged Cdc9 with the indicated Dnl4 mutations and the *GAL1*-cs allele were pre-grown to log phase in YPA-Glycerol (**A, B**) or post-diauxic phase (**C, D**) and treated with 2% galactose to induce HO expression and DSB formation. (**A**) and (**C**) show the fraction of intact *GAL1*-cs HO cut sites and thus the extent of DSB formation and repair over time as determined by flanking PCR. (**B**) and (**D**) show the corresponding enrichment of 13Myc-Cdc9 at the *GAL1*-cs DSB as determined by ChIP from the same samples as (A) and (C), respectively. ‘No ab’ indicates background signal obtained without antibody addition during immunoprecipitation. Results are the mean ± standard deviation of two independent experiments.

A possible explanation for the above results is that Cdc9/DNA ligase I, the only other DNA ligase in *S. cerevisiae*, catalyzes the residual NHEJ when Dnl4 is present but inactive. It is difficult to perform NHEJ assays in *cdc9* yeast as it is an essential gene, so to begin to assess its potential role in residual NHEJ we performed Cdc9 ChIP. A first experiment revealed the appearance of Cdc9 at the *GAL1*-cs DSB after one hour of galactose induction and its disappearance following glucose addition (Figure 7A,B). This pattern was very similar to Dnl4 (Figure 3), although the level of Cdc9 enrichment was lower. To make the signal more sustained we repeated the experiment by pre-growing the cells to post-diauxic phase and did not add glucose at one hour, which was expected to prevent 5′ resection and promote residual NHEJ according to results above. DSB induction was delayed in this protocol (Figure 7C), but Cdc9 enrichment was again observed that now persisted for four hours (Figure 7D). Given the lower DSB levels, this pattern suggests a more robust Cdc9 enrichment in post-diauxic than log phase cells. Controls lacking the anti-Myc ChIP antibody confirmed the specificity of this result, for which DSB induction in fact lowered the DSB locus signal relative to the *ACT1* control locus, perhaps due to reduced non-specific binding of the DSB fragment resulting from local chromatin alterations upon DSB induction (Figure 7D) [32]. Finally, a pattern emerged in post-diauxic cells wherein Cdc9 signals were lowest in the complete absence of Dnl4 and highest in the presence of Dnl4-K466A (Figure 7D). This trend was again not predicted by the higher levels of DSB formation in the *dnl4Δ* strain, suggesting a promotion of Cdc9 retention by Dnl4 in post-diauxic cells.

## Discussion

The central findings of this work are that (i) catalytically inactive yeast Dnl4/DNA ligase IV can promote imprecise NHEJ when it persists at DSBs, (ii) Cdc9/DNA ligase I is present at DSBs at times consistent with it catalyzing the observed residual NHEJ in place of Dnl4, (iii) these phenomenon and DSB 5′ resection are influenced by the metabolic state of the cells, and (iv) 5′ resection efficiency is reduced by c-NHEJ activity but not by the presence of Dnl4 protein at a DSB, suggesting an active process that removes NHEJ proteins and initiates 5′ resection.

### Dnl4 Supports Mutagenic NHEJ Independently of Its Catalytic Activity

We made mutations in universally conserved Dnl4 residues that have been shown to create step-specific catalytic blocks in other ATP-dependent DNA ligases [20], [21]. Dnl4-K282R could not auto-adenylate, as expected, but we also observed severely impaired capacity of catalytic step 1 *in vitro* with Dnl4-D284A and Dnl4-K466A (Figure 2A) and commensurate undetectable auto-adenylation *in vivo* with Dnl4-K466A (Figure S4). This pattern of results emphasizes the integrated nature of the DNA ligase active site. Consistently, Dnl4-K282R, -D284A and -K466A mutants were all severely deficient in c-NHEJ *in vivo* in several assays (Figures 2–4). No mutant acted as a dominant negative (Figure 5B), despite the fact that all bound Lif1 (Figure 2C,D) and accumulated even more than wild-type at DSBs (Figure 3). Importantly, DSB recruitment must be distinguished from fully productive binding to DNA termini, which likely requires ligase auto-adenylation [5], [20].

Given the expected severe effect of Dnl4 catalytic mutation on ligase activity and c-NHEJ, it was striking that these same mutants supported residual NHEJ activity in the suicide deletion and *ILV1*-cs assays well above that observed in strains lacking Dnl4 (Figures 5 and 6). We considered a number of properties of the suicide deletion assay to account for this residual repair. The phenomenon did not depend on the use of two DSBs since a difference in residual NHEJ was also observed in the single-DSB *ILV1*-cs next-generation sequencing experiment (Figure 6). In contrast, increased survival due to residual suicide deletion NHEJ was observed only when cells were pre-grown to post-diauxic/early stationary phase (Figure 5). With the *ILV1*-cs assay, the detection of Dnl4 protein-dependent residual NHEJ entailed a growth protocol that promoted the recovery of imprecise joints by prolonged HO expression in the context of limited 5′ resection due to the inability of the cells to ferment galactose.

Despite the technical differences in execution, the suicide deletion and *ILV1*-cs assays led to the same conclusion that a substantial extent of imprecise NHEJ is preserved with catalytically defective Dnl4 but lost in the absence of Dnl4 protein. Thus, the form of NHEJ supported by Dnl4 protein was more mutagenic than the proper c-NHEJ catalyzed by wild-type Dnl4. This observation is reminiscent of findings that Dnl4 participates in some microhomology mediated end joining (MMEJ) through a poorly understood mechanism [33] and of mammalian studies documenting the mutagenic potential of DNA ligase IV-independent NHEJ [16], [34]. It also has implications for the human ligase IV syndrome in which mutations such as R278H, Q280R, and H282L (motif I) and G468E, G469E (motif V) are hypomorphic despite the fact that they severely alter the ATP binding properties of the enzyme [35], [36]. This pattern is very similar to the yeast mutants studied here and suggests the possibility for affected humans that inactive ligase might accumulate at a DSB and support mutagenic NHEJ.

### Cdc9 as a Candidate Imprecise NHEJ Ligase

Joint analysis unequivocally established that residual repair observed with Dnl4 catalytic mutants occurred by NHEJ (Figure 6 and S3 and Table 1), demonstrating that NHEJ can be catalyzed by a ligase other than Dnl4. We cannot entirely rule out that residual repair was catalyzed by some Dnl4 mutants, but K282R is incapable of becoming adenylated (Figure 2A) and we found no evidence to support adenylation and c-NHEJ ligation by K466A (Figures 2 and S4). In contrast, Cdc9 was recruited to DSBs *in vivo* in a time frame consistent with its participation in NHEJ and in a manner that was enhanced by Dnl4 protein (Figure 7). The difference in the imprecise joint profile obtained with wild-type and Dnl4-K466A (Figures 6B and S6) provides further indirect support for the participation of a different ligase in the residual imprecise NHEJ. Dnl4-independent NHEJ is not especially surprising since even some precise NHEJ occurs in the complete absence of Dnl4 [25] and can become quite efficient as overhang lengths increase [37]. Moreover, DNA ligase III, which is missing from yeast, and to a lesser extent DNA ligase I have been shown to catalyze alt-NHEJ in higher eukaryotes [14], [15]. The key finding here is that use of an alternative ligase leading to especially imprecise NHEJ can be stimulated by the presence of Dnl4 protein at a DSB, to the point that one must consider whether many mutagenic NHEJ events are catalyzed by DNA ligase I/III even when DNA ligase IV is present, an idea supported by mammalian studies [14].

The exact mechanism by which Dnl4 protein might promote imprecise NHEJ by Cdc9 is unknown, but numerous observations suggest that it is the c-NHEJ complex that is responsible even if the events are not catalyzed by Dnl4. All tested c-NHEJ proteins accumulate at DSBs in strains bearing Dnl4 catalytic mutations in a manner that parallels Dnl4 itself (Figure 3), so all are likely present when residual NHEJ occurs. Further, it is well established for suicide deletion and other assays that loss of Ku, Lif1 and MRX alone leads to a severe loss of all NHEJ, including imprecise events, in the same fashion as *dnl4*Δ [30]. In the one case where we could specifically test a role of another c-NHEJ protein, we indeed found that the residual NHEJ observed in the absence of Dnl4 catalytic activity depends on Nej1 (Figure 5D). The mode of mutagenic NHEJ we describe here thus appears to be distinct from yeast MMEJ [33] or alt-NHEJ more generally, which are inhibited by the c-NHEJ assembly containing Ku [2]. Instead, Dnl4 protein likely promotes assembly of the c-NHEJ complex at the DSB, as has been proposed [11], for subsequent action not only by Dnl4 but also Cdc9 and likely many other enzymes. This concept is consistent with electron microscopy data that DNA ligase IV can execute end bridging in conjunction with Ku [38], as well as mammalian studies suggesting that DNA ligase IV acts non-catalytically at DSBs in the early stage of NHEJ [39], perhaps by supporting filament formation seen with XRCC4/XLF [40], [41].

### Dnl4 Indirectly Inhibits DSB Resection

We monitored the flux of DSBs into the competing NHEJ and HR pathways to determine whether resection is inhibited by Dnl4 as has been suggested [11]. The results demonstrate that resection was inhibited by Dnl4 only because it catalyzed NHEJ that removed DSBs from the potential resection pool (Figure 4). DSBs that failed NHEJ were shunted to 5′ resection with equal efficiency whether the failure was created by loss of Dnl4 or by a Dnl4 point mutant that accumulated at the DSB. This behavior is explained by the observation that both wild-type and mutant Dnl4 (Figure 3), as well as Cdc9 (Figure 7), were efficiently removed from persistent DSBs. Although the mechanistic sequence remains to be established, these results support a model in which NHEJ protein removal is an active process initiated after an initial time period of end preservation to permit NHEJ [2], [27], [42]. The notion that it is an active process is supported by the observation that the utilizable carbon source, and by inference the energy status, had a large influence on the cell's ability to engage in 5′ resection (Figure 4) and ligase removal (Figure 7).

The combined observations on residual NHEJ activity and resection emphasize the impact that cell state, including both cell cycle stage and metabolic influences, has on DSB repair outcomes. Such ideas are relevant to human DNA repair since most cells in an adult mammal are in the G0 state and under careful metabolic control that is disrupted in cancer [43]. Indeed, recent studies demonstrate a strong influence of cell state on mutagenic NHEJ outcomes not unlike those observed here [44]. These insights also suggest possible unintended mutagenic consequences of DNA ligase IV inhibitors [45], agents which might lead to accumulation of catalytically ineffective c-NHEJ complexes at DSBs in a manner similar to the mutants studied here.

## Materials and Methods

### Protein Structural Analysis

Protein structural analysis was performed using The PyMOL Molecular Graphics System, Schrödinger, LLC.

### Yeast Growth and Manipulation

Yeast strains used for NHEJ and chromatin immunoprecipitation (ChIP) assays (Table S1) were isogenic derivatives of BY4741 [46] as previously described [27], [28]. Point mutations were constructed in the native *DNL4* gene using a pop-in/pop-out method [29]. L750* truncation was created as a stop codon after the indicated residue. All mutant alleles were confirmed by sequencing. Yeast were grown at 30°C in either rich medium containing 1% yeast extract, 2% peptone and 40 µg/ml adenine (YPA) or synthetic defined (SD) medium with either 2% glucose, 2% galactose or 3% glycerol as the carbon source [27], [28].

### Yeast Two-Hybrid

The two hybrid indicator strain PJ69-4a/α, vectors pODB2 and pOAD, and full-length and truncated Dnl4 and Lif1 derivatives have been described [29], [47]. Dnl4 point mutant derivatives were made by transferring mutant chromosomal alleles into plasmids by gap repair [29]. Dnl4-Lif1 interaction was monitored by mating to strains carrying Lif1 constructs and spotting overnight cultures to plates lacking either histidine or adenine followed by 3 and 5 days growth, respectively.

### Plasmid Recircularization Assay

pRS316 [46] was cut with *Nco*I to create a 5′ overhanging DSB within its *URA3* marker. pK1827 [26] was cut with *Kpn*I to create a 3′ overhanging DSB within its *ADE2* marker, leaving its *URA3* marker intact. Each cut plasmid (100 ng) was co-transformed into YW1228 derivatives along with 10 ng of supercoiled *HIS3*-marked pRS413 [46], as previously described [28]. c-NHEJ efficiency is reported as the ratio of Ura^+^ (pRS316) or Ura^+/^Ade^+^ colonies (pK1827) to His^+^ colonies.

### Suicide Deletion Assay

The suicide deletion assay for monitoring NHEJ was previously described [26], [29], [30]. Here all strains (Table S1) bore the *ade2*::SD2-::*STE3*-*MET15* allele for which HR is not possible. Cells were pre-grown either to stationary phase for 2 days in SD medium, our standard protocol [26], or to log phase overnight in YPA-Glycerol. NHEJ was scored as colony counts on galactose plates divided by counts on parallel glucose control plates. The nature of NHEJ events in Ade^+^ colonies was determined by mating to YW2083 carrying the I-*Sce*I expression plasmid pTW334 [26]. If precise NHEJ had created an intact I-*Sce*I site in the suicide deletion strain, it is recleaved and undergoes HR with the YW2083 *ade2*-M7 allele to give Ade^−^/red diploids. Ade^−^ suicide deletion colonies were assessed by first ensuring an isolate was Met^−^, and thus had deleted *STE3*-*MET15*, and then attempting to amplify a 1.2 kb PCR product symmetrically flanking the expected DSB junction. No product indicated a large deletion. Inferred imprecise NHEJ alleles were finally sequenced.

### Chromosomal DSB Induction

We previously described the *GAL1*-cs and *ILV1*-cs strains for creating single-site chromosomal DSBs [27]. In them, HO is expressed from the native chromosomal *GAL1* promoter so that the strain is rendered Gal^−^. HO cut sites are present in either the same *GAL1* promoter that expresses HO (*GAL1*-cs) or in the native *ILV1* promoter (*ILV1*-cs). DSB induction and α-factor G1 arrest were as described [27].

### Epitope Tagging and ChIP

*GAL1*-cs strains bore a 13Myc tag on the C-terminus of the native chromosomal *DNL4, CDC9*, *LIF1* and *YKU80* genes as previously described [27]. ChIP assays and parallel monitoring of DSB formation and repair were performed as described [27] except that quantitative PCR rather than competitive PCR was used to monitor Dnl4 binding to the DSB site, with fold enrichment determined from the Ct difference between *GAL1* and the *ACT1* control gene.

### Immunoprecipitation and Adenylation Assays

*GAL1*-cs strains were grown overnight in YPA-Dextrose. Lysates were prepared from 1 g wet cell pellet in lysis buffer (10 mM Tris pH 7.5, 150 mM NaCl, 1 mM MgCl_2_, 2 mM CaCl_2_, 10% glycerol, and Roche complete mini tablet) using zirconia bead lysis followed by centrifugation at 15,000× g. Dnl4 was immunoprecipitated by overnight incubation with 80 µl anti-Myc conjugated agarose beads (Sigma). Samples were washed 3 times with lysis buffer, divided into two aliquots, and pre-incubated with 1 ml AMP buffer (60 mM Tris pH 8.0, 10 mM MgCl_2_, 2 mM DTT, and Roche complete mini tablet) with or without 0.5 mM sodium pyrophosphate for 15 min at room temperature. Beads were washed with 1 ml AMP buffer before addition of 15 µCi α-^32^P ATP to the residual volume for 30 min at room temperature. Beads were washed twice before boiling and electrophoresis on a 7.5% SDS-PAGE gel and transfer to nitrocellulose. The same membrane was used to obtain a phosphorimager screen exposure followed by Western blotting using anti-Myc antibody with detection by a LiCor Odyssey scanner. Results are expressed as the ratio of phosphorimager to Western blot signal, setting the wild-type Dnl4 sample with pyrophosphate treatment to 100%.

### Resection Monitoring

Genomic DNA was extracted after galactose induction in the *ILV1*-cs system, digested with *Eco*RI and *Nco*I, and subjected to Southern blotting as previously described [27]. Results are expressed as a ratio of the intensity of the intact (2.6 kb) or HO-cut (1.1 kb) *ILV1* fragment to that of the *APN1* control fragment (3.5 kb), normalized to the ratio at either 0 or 60 minutes of induction, as indicated.

### Recombination Assay

*ILV1*-cs yeast were transformed with pRS416 containing a 960 bp homologous *ILV1* donor fragment. Cells were plated to glucose plates after varying times of DSB induction in galactose to determine cell survival relative to untreated cells.

### Next Generation Sequencing

*ILV1*-cs yeast were pre-grown overnight in YPA-Glycerol. Genomic DNA was harvested before and 24 hours after galactose induction and subjected to PCR using primers flanking the *ILV1* HO DSB site that additionally bore unique 6-base sequence indexes for each input sample and a 5′ extension matching Illumina paired-end sequencing adapters (5′-ACACTCTTTCCCTACACGACGCTCTTCCGATCTxxxxxxAGGGCAAAAAGAAAAAGCGCAG and 5′-CTCGGCATTCCTGCTGAACCGCTCTTCCGATCTxxxxxxGTTTTATCAAGGAAGGTGACA, where “xxxxxx” was replaced with specific index sequences). After 15 cycles of amplification, products were purified and subjected to an additional 16 PCR cycles using Illumina paired-end second-round primers. Products from all samples were pooled, 50% of a PhiX control library was added to facilitate cluster calling, and the mixture subjected to Illumina HiSeq 100 bp paired-end sequencing at the University of Michigan DNA Sequencing Core. Only forward reads crossed the *ILV1* HO cut site and were used for further analysis. Reads matching PhiX were removed using Bowtie [48]. Custom Perl scripts (File S2) assigned the remaining reads to the input samples based on the index and counted the number that corresponded to the intact *ILV1* HO cut site or any one of the possible blunt, microhomology, or insertion-mediated joints supported by the input DSB ends, allowing up to two inserted bases at the junction and one mismatched read base. Reads corresponding to more than one joint were called ambiguous. Joint counts were input into DESeq [31] as is, or expressed as a fraction of the total *ILV1* read counts for graphical comparisons.

### Dnl4 Mass Spectrometry

pTW644 (a kind gift Patrick Sung) expresses Dnl4 and His6-tagged Lif1 from divergent *GAL1/10* promoters. Dnl4-K466A was introduced into this plasmid by PCR amplification and ligation into *BamH*I/*Sal*I sites and confirmed by sequencing. Plasmids were transformed into YW1230 for suicide deletion analysis as described above and into protease-deficient YW2189 for protein purification. Yeast were grown to log phase in 1000 ml of YPA-Glycerol and expression induced by addition of 2% galactose for 4 hours. 5 g of cells were harvested, resuspended in buffer A [28], and lysed using zirconium beads (Biospec). The cleared lysate was incubated with 0.3 ml of nickel-NTA agarose beads (Qiagen) in the presence of 10 mM imidazole for 1 h at 4°C. The beads were washed three times with buffer A containing 20 mM imidazole and boiled in 1X SDS loading buffer. Following 10% SDS-PAGE and Novex colloidal blue staining (Invitrogen), Dnl4 bands were excised and subjected to trypsin digestion. Resulting peptides were resolved on a nano-capillary reverse phase column (Picofrit column, New Objective) using a 1% acetic acid/acetonitrile gradient, introduced into a linear ion-trap mass spectrometer (LTQ Orbitrap XL, ThermoFisher), and searched against the *S. cerevisiae* protein database using X!Tandem/TPP [49]. Oxidation of methionine (+15.9949 Da), carbamidomethylation of cysteines (+57.0214 Da), and AMP-lysine (+329.0525 Da) were allowed modifications. Precursor and fragment mass tolerance were 50 ppm and 0.8 Da, respectively. Matches to Dnl4 with a ProteinProphet probability of >0.9 (fdr <2%) were used to determine the adenylation state of K282-containing peptides.

## Supporting Information

Figure S1 DSB resection is inefficient in the absence of a fermentable carbon source. Analysis of DSB resection at the *ILV1*-cs allele was monitored by Southern blotting in *gal1* yeast complemented with vector (Gal−) or p*GAL1* (Gal+) without the addition of glucose at 60 min, similar to Figure 4A. *gal1* yeast cannot metabolize the galactose added to induce HO expression and DSB formation, but glycerol was present as a carbon and energy source throughout the experiment. (A) The ratio of the HO-uncut band to the *APN1* control was normalized to the ratio at time 0 to allow monitoring of DSB formation and repair by NHEJ. (B) The ratio of the HO-cut band to the *APN1* control was normalized to the ratio at 60 min when DSB formation was maximal. Results are the mean ± standard deviation of two independent experiments. DSBs were formed but only very slowly resected in *gal1* yeast, even at the 180 min time point when resection was nearly complete in *GAL1* strain.
(PDF)

Figure S2 DSB repair by homologous recombination in Dnl4 catalytic mutant strains. *ILV1*-cs yeast bearing the indicated Dnl4 alleles, with (A) and without (B) a homologous *ILV1* donor fragment on a plasmid, were pre-grown to log phase in YPA-Glycerol and treated with 2% galactose for the indicated times to induce a DSB. Cells were then plated to glucose and survival was determined relative to untreated cells. Survival in wild-type with the homologous donor reflects DSB repair by both HR and c-NHEJ. The *dnl4* mutants are all defective in c-NHEJ so that survival with the donor reflects equivalent rates of HR. Results are the mean ± standard deviation of three independent experiments. EV, empty vector.
(PDF)

Figure S3 Imprecise joints in the suicide deletion assay. Sequenced imprecise DSB repair junctions from colonies formed in the suicide deletion assay. (A) Ade^+^/white colonies that did not recleave the allele upon re-introduction of I-*Sce*I (see Table 1). (B) Ade^−^/red colonies. In each, the sequence at the top left is the product of precise suicide deletion NHEJ, equivalent to an I-*Sce*I cut site. Bold type shows the location of the 4-base 3′ overhang. Subsequent entries show recovered imprecise joints and the number of times they were observed in different strains. Underlined bases are microhomologies, dashes indicate deleted bases, and lower case indicates inserted bases.
(PDF)

Figure S4 Impaired Dnl4-K466A auto-adenylation *in vivo*. (A) Protein gel showing expression of the Dnl4-Lif1 complex in strains transformed with plasmids bearing the indicated components. (B) NHEJ efficiency in the suicide deletion assay in yeast transformed with the same plasmids as in (A). Results are the mean ± standard deviation of three independent experiments. (C) Dnl4-K466A adenylation status *in vivo*. TLHDDFLVEEK is the fully cleaved and unadenylated tryptic peptide ending at K282. TLHDDFLVEEKMDGER and TLHDDFLVEEK*MDGER are not cleaved at K282, with K* indicating K282 adenylation. Results are from multiple lanes and mass spectrometry runs from two biological replicates for each of wild-type and Dnl4-K466A. Note that K466 is not contained within the peptides shown.
(PDF)

Figure S5 Description of the joint identifiers used in next-generation sequencing. HO-induced DSB formation in the *ILV1*-cs system is shown at the top. The HO 3′ overhangs are in red and the surrounding *ILV1* promoter sequence is in magenta. In the coded joint identifiers, “D” indicates the number of base pairs lost or gained in the joint overall, “M” indicates the number of microhomologous base pairs at the repair junction, “L” indicates the number of base pairs deleted from the left side of the DSB, as measured from the most distal base of the overhang, “R” similarly indicates the number of base pairs deleted from the right side, and “I” indicates any non-templated insertion nucleotides at the repair junction, read from the top strand. Common joint types are shown as examples. +CA and −ACA joint designations reflect the nomenclature used by Moore and Haber [50].
(PDF)

Figure S6 *ILV1*-cs next-generation DESeq analysis. MA plots of the output of DESeq runs that compared (A) the two wild-type 24-hour replicates to each other, (B) the two K466A 24-hour replicates to each other, and (C) the wild-type 24-hour replicates to the K466A 24-hour replicates. Data points represent individual joint types. Red points highlight joints with a Bonferroni-adjusted p-value<0.005. Results demonstrate a much higher concordance between replicates of the same sample than between wild-type and K466A.
(PDF)

File S1 *ILV1*-cs next-generation sequencing results. Excel file containing data on the precise and major imprecise joints observed in the *ILV1*-cs next-generation sequencing experiments. A major imprecise joint was present in ≥0.1% of the reads of any individual sample. (A) Worksheet “Counts” tabulates the read counts for each sample replicate for the observed joints. Joint assignments were made as described in Materials and Methods. Joints are sorted by the frequency of occurrence. Columns are labeled in format “sample_timepoint_replicate”. Joint identifiers are described in Figure S5. One read (row 20) did not match an expected joint and is listed as the observed read sequence. (B) Worksheet “Sequences” shows, for each observed joint, the joint structure with microhomologous bases in the middle, as well as the joint sequences without added spaces. Joints are in the same order as in (A). (C) Worksheet ‘DESeq” shows the output of the DESeq analysis performed on the wt and K466A 24-hour data. “baseMean” is the read count after normalization to allow for inter-sample comparisons, “padj” is the Bonferroni-adjusted p-value of the joint, comparing wt replicates to K466A replicates, and “K466A effect” is the direction of statistically significant K466A mutant effects (p<0.005). Joints are sorted by padj.
(XLS)

File S2 Perl scripts used in *ILV1*-cs next-generation sequencing. Zip file containing three Perl scripts used to assign sequence reads to joint identifiers. See scripts for details.
(ZIP)

Table S1 Genotype of yeast strains used in this study.
(DOCX)
