# Supplementary figures and images for: Saccharomyces cerevisiae DNA Ligase IV Supports Imprecise End Joining Independently of Its Catalytic Activity
Source: PLoS Genet. 2013 Jun 27;9(6):e1003599. doi: 10.1371/journal.pgen.1003599 (PMC3694833; doi:10.1371/journal.pgen.1003599)

Figure S1

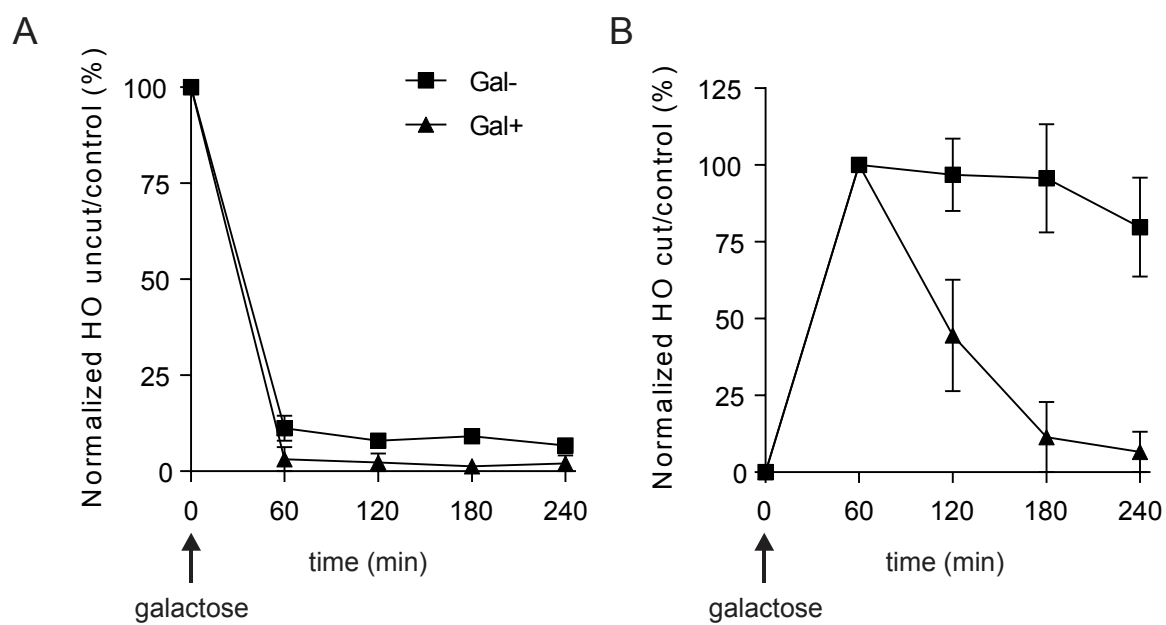

Supplement: Figure S1 — DSB resection is inefficient in the absence of a fermentable carbon source. Analysis of DSB resection at the ILV1-cs allele was monitored by Southern blotting in gal1 yeast complemented with vector (Gal−) or pGAL1 (Gal+) without the addition of glucose at 60 min, similar to Figure 4A. gal1 yeast cannot metabolize the galactose added to induce HO expression and DSB formation, but glycerol was present as a carbon and energy source throughout the experiment. (A) The ratio of the HO-uncut band to the APN1 control was normalized to the ratio at time 0 to allow monitoring of DSB formation and repair by NHEJ. (B) The ratio of the HO-cut band to the APN1 control was normalized to the ratio at 60 min when DSB formation was maximal. Results are the mean ± standard deviation of two independent experiments. DSBs were formed but only very slowly resected in gal1 yeast, even at the 180 min time point when resection was nearly complete in GAL1 strain. (PDF) [file pgen.1003599.s001.pdf]

Figure S2

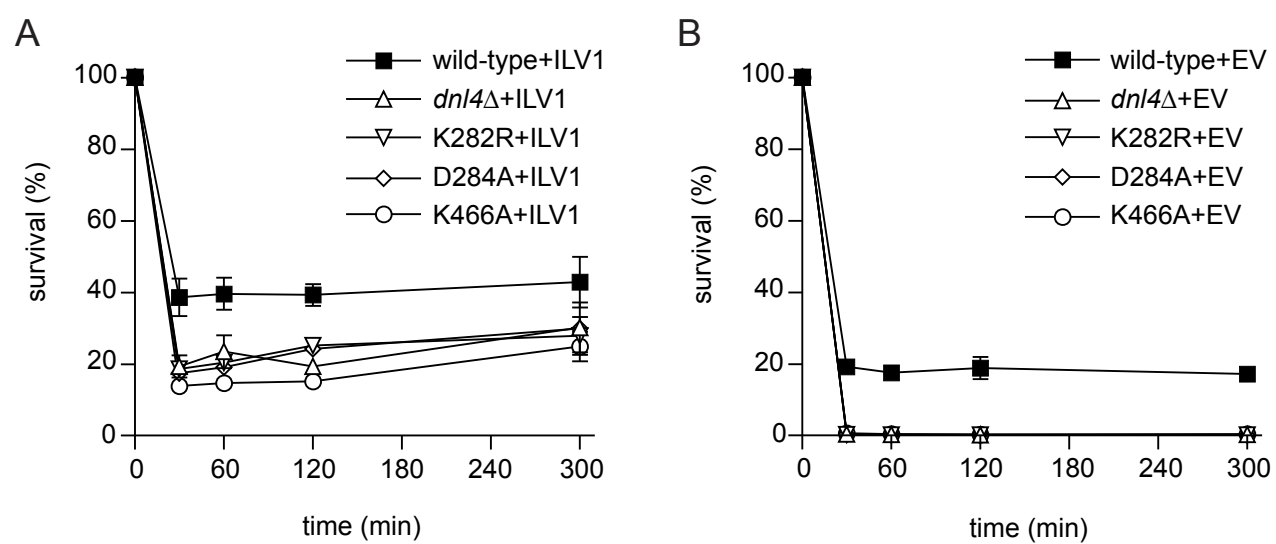

Supplement: Figure S2 — DSB repair by homologous recombination in Dnl4 catalytic mutant strains. ILV1-cs yeast bearing the indicated Dnl4 alleles, with (A) and without (B) a homologous ILV1 donor fragment on a plasmid, were pre-grown to log phase in YPA-Glycerol and treated with 2% galactose for the indicated times to induce a DSB. Cells were then plated to glucose and survival was determined relative to untreated cells. Survival in wild-type with the homologous donor reflects DSB repair by both HR and c-NHEJ. The dnl4 mutants are all defective in c-NHEJ so that survival with the donor reflects equivalent rates of HR. Results are the mean ± standard deviation of three independent experiments. EV, empty vector. (PDF) [file pgen.1003599.s002.pdf]

Figure S4

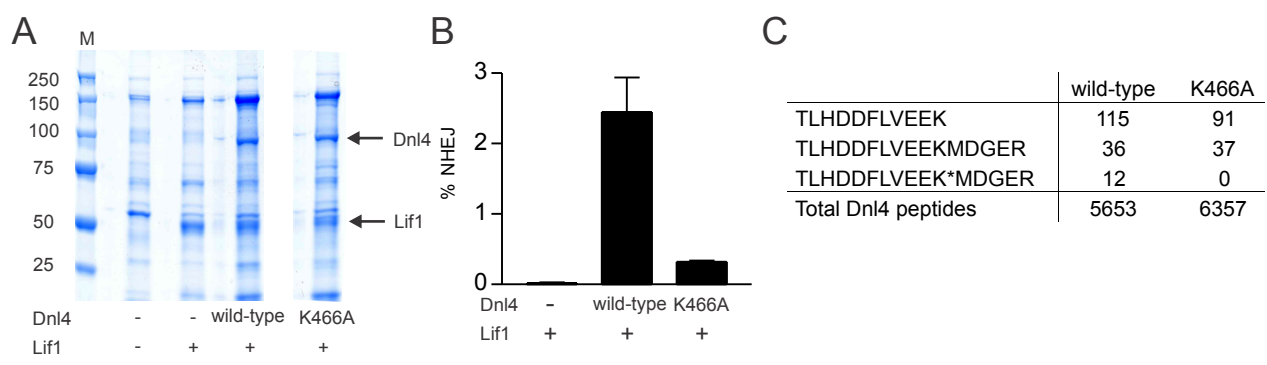

Supplement: Figure S4 — Impaired Dnl4-K466A auto-adenylation in vivo. (A) Protein gel showing expression of the Dnl4-Lif1 complex in strains transformed with plasmids bearing the indicated components. (B) NHEJ efficiency in the suicide deletion assay in yeast transformed with the same plasmids as in (A). Results are the mean ± standard deviation of three independent experiments. (C) Dnl4-K466A adenylation status in vivo. TLHDDFLVEEK is the fully cleaved and unadenylated tryptic peptide ending at K282. TLHDDFLVEEKMDGER and TLHDDFLVEEK*MDGER are not cleaved at K282, with K* indicating K282 adenylation. Results are from multiple lanes and mass spectrometry runs from two biological replicates for each of wild-type and Dnl4-K466A. Note that K466 is not contained within the peptides shown. (PDF) [file pgen.1003599.s004.pdf]

Figure S6

**(A)** wt-1 vs. wt-2

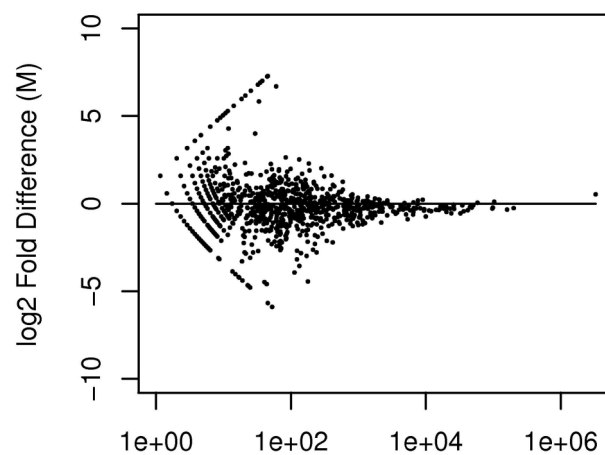

**(B)** K466A-1 vs. K466A-2

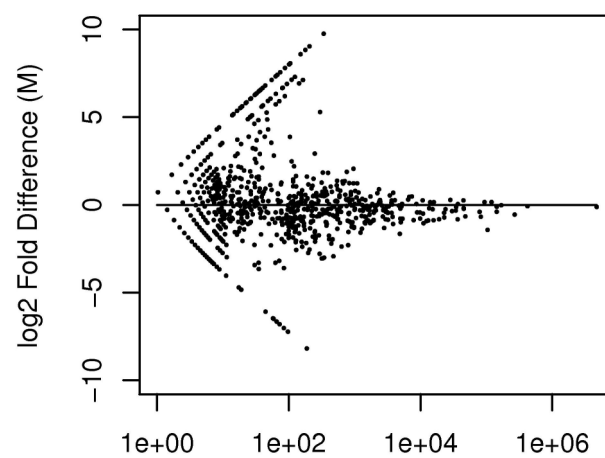

**(C)** wt vs. K466A

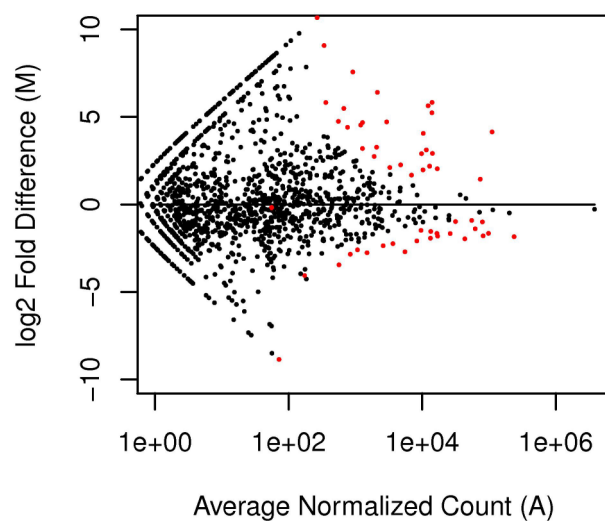

Supplement: Figure S6 — ILV1-cs next-generation DESeq analysis. MA plots of the output of DESeq runs that compared (A) the two wild-type 24-hour replicates to each other, (B) the two K466A 24-hour replicates to each other, and (C) the wild-type 24-hour replicates to the K466A 24-hour replicates. Data points represent individual joint types. Red points highlight joints with a Bonferroni-adjusted p-value<0.005. Results demonstrate a much higher concordance between replicates of the same sample than between wild-type and K466A. (PDF) [file pgen.1003599.s006.pdf]
