# Supplementary material for: Saccharomyces cerevisiae DNA Ligase IV Supports Imprecise End Joining Independently of Its Catalytic Activity
Source: PLoS Genet. 2013 Jun 27;9(6):e1003599. doi: 10.1371/journal.pgen.1003599 (PMC3694833; doi:10.1371/journal.pgen.1003599)
Supplement: Figure S3 — Imprecise joints in the suicide deletion assay. Sequenced imprecise DSB repair junctions from colonies formed in the suicide deletion assay. (A) Ade+/white colonies that did not recleave the allele upon re-introduction of I-SceI (see Table 1). (B) Ade−/red colonies. In each, the sequence at the top left is the product of precise suicide deletion NHEJ, equivalent to an I-SceI cut site. Bold type shows the location of the 4-base 3′ overhang. Subsequent entries show recovered imprecise joints and the number of times they were observed in different strains. Underlined bases are microhomologies, dashes indicate deleted bases, and lower case indicates inserted bases. (PDF) [file pgen.1003599.s003.pdf]

Figure S3

A

GATAAACGCGTGTATTACCCTG **TTAT** CCCTAGCGTCAGATCCTCTAGAA	**WT**	**K282R**	**D284A**	**K466A**
GATAAACGCGTGTATTA -----CCCT----AGCGTCAGATCCTCTAGAA	6/10	5/10	4/10	4/10
GATAAAC ----------------GCGT--------- CAGATCCTCTAGAA	3/10	1/10	4/10	1/10
GATAAACGCGTGTATTACCCTGT TA------ GCGTCAGATCCTCTAGAA	1/10			
GATAAACGCGTGTA ---------TATCCCTAGCGTCAGATCCTCTAGAA		2/10		1/10
GATAAACGCGTGTATTACCC ---TATCCCTAGCGTCAGATCCTCTAGAA		1/10	1/10	2/10
GATAAACGCGTGTATTACCCTG --------- GCGTCAGATCCTCTAGAA		1/10		
GATAAACGCGT GT--------- TTATCCCTAGCGTCAGATCCTCTAGAA				1/10
GATAAACGCGTGTATTACC ---a---CCCTAGCGTCAGATCCTCTAGAA			1/10	
GATAAACGCGTGTATTACCCTGTTAaCCCTAGCGTCAGATCCTCTAGAA				1/10

B

GATAAACGCGTGTATTACCCTG **TTAT** CCCTAGCGTCAGATCCTCTAGAA	**WT**	**K282R**	**D284A**	**K466A**
GATAAACGCGTGTATTACCCTGtTTATCCCTAGCGTCAGATCCTCTAGAA	2/5			
GATAAACGCGTGTATTACCC ----ATCCCTAGCGTCAGATCCTCTAGAA	1/5		1/3	
GATAAACGCGTGTA --------TTATCCCTAGCGTCAGATCCTCTAGAA	1/5			2/5
GATAAACGCGTGTATTACCCTGTTAT -CCTAGCGTCAGATCCTCTAGAA	1/5			
GATAAACGCGTG ----------- TATCCCTAGCGTCAGATCCTCTAGAA		2/2		
GATAAACGCGTGTATTA -CCTGTTATCCCTAGCGTCAGAT CCTCTAGAA			1/3	
GATAAACGCGTGTATTACCCTGTTA -CCCTAGCGTCAGATCCTCTAGAA			1/3	
GATAAACGCGTGTAT TACCCTGT TATatCCCTAGCGTCAGATCCTCTAGAA				2/5
GATAAACGCGTGTATTACCCTG -TATCCCTAGCGTCAGATCCTCTAGAA				1/5
