# Supplementary material for: Saccharomyces cerevisiae DNA Ligase IV Supports Imprecise End Joining Independently of Its Catalytic Activity
Source: PLoS Genet. 2013 Jun 27;9(6):e1003599. doi: 10.1371/journal.pgen.1003599 (PMC3694833; doi:10.1371/journal.pgen.1003599)
Supplement: Figure S5 — Description of the joint identifiers used in next-generation sequencing. HO-induced DSB formation in the ILV1-cs system is shown at the top. The HO 3′ overhangs are in red and the surrounding ILV1 promoter sequence is in magenta. In the coded joint identifiers, “D” indicates the number of base pairs lost or gained in the joint overall, “M” indicates the number of microhomologous base pairs at the repair junction, “L” indicates the number of base pairs deleted from the left side of the DSB, as measured from the most distal base of the overhang, “R” similarly indicates the number of base pairs deleted from the right side, and “I” indicates any non-templated insertion nucleotides at the repair junction, read from the top strand. Common joint types are shown as examples. +CA and −ACA joint designations reflect the nomenclature used by Moore and Haber [50]. (PDF) [file pgen.1003599.s005.pdf]

Figure S5

HO-induced DSB

CAAATTTGGAATCGCTTTTAGTTTCAGCTTTCCGCAACA GTATAATTTTATAAACCCTGATTTGGAATCGCATA
GTTTAAACCTTAGCGAAAATCAAAGTCGAAAGGCG TTGTCATATTAAAATATTTGGGACTAAACCTTAGCGTAT

ILV1prm overhang

coded name	joint type	joint sequence
D+0_M4_L0_R0	precise	TTTTAGTTTCAGCTTTCCGCAACAGTATAATTTTATAAACCCTG<br>AAAATCAAAGTCGAAAGGCGTTGTCATATTAAAATATTTGGGAC<br>microhomology
D-4_M0_L4_R4	resected blunt	TTTTAGTTTCAGCTTTCCGCGTATAATTTTATAAACCCTG<br>AAAATCAAAGTCGAAAGGCGCATATTAAAATATTTGGGAC
D+2_M1_L0_R1	+CA	templated fill-in<br>TTTTAGTTTCAGCTTTCCGCAACACAGTATAATTTTATAAACCCTG<br>AAAATCAAAGTCGAAAGGCGTTGTGTCATATTAAAATATTTGGGAC
D-3_M2_L3_R2	-ACA	TTTTAGTTTCAGCTTTCCGCAGTATAATTTTATAAACCCTG<br>AAAATCAAAGTCGAAAGGCGTCATATTAAAATATTTGGGAC
D+4_M0_L0_R0	fill-in blunt	TTTTAGTTTCAGCTTTCCGCAACAAACAGTATAATTTTATAAACCCTG<br>AAAATCAAAGTCGAAAGGCGTTGTTTGTCATATTAAAATATTTGGGAC
D+6_M0_L0_R0_I(AC)	fill-in plus	non-templated fill-in<br>TTTTAGTTTCAGCTTTCCGCAACAACAACAGTATAATTTTATAAACCCTG<br>AAAATCAAAGTCGAAAGGCGTTGTTGTTGTCATATTAAAATATTTGGGAC

D= delta, the number of bases lost/gained

M= the number of bases of microhomology

L= the number of bases deleted on the left overhang

R= the number of bases deleted on the right overhang

I= insertional nucleotides
