# Supplementary material for: Saccharomyces cerevisiae DNA Ligase IV Supports Imprecise End Joining Independently of Its Catalytic Activity
Source: PLoS Genet. 2013 Jun 27;9(6):e1003599. doi: 10.1371/journal.pgen.1003599 (PMC3694833; doi:10.1371/journal.pgen.1003599)
Supplement: Table S1 — Genotype of yeast strains used in this study. (DOCX) [file pgen.1003599.s009.docx]

| **Strain** | **Genotype** |
| --- | --- |
| YW1228 | *MATα* *ade2*::*SD2*-::*STE3*-*MET15 his3*∆1 *leu2*∆0 *met15*∆0 *ura3*∆0 |
| YW1230 | YW1228 *dnl4*∆::*kanMX4* |
| YW2042 | YW1228 *dnl4*-K282R |
| YW2043 | YW1228 *dnl4*-D284A |
| YW2044 | YW1228 *dnl4*-K466A |
| YW2051 | YW1228 *dnl4*-L750* |
| YW2304 | YW1228 *nej1*::*kanMX4* |
| YW2305 | YW2042 *nej1*::*kanMX4* |
| YW2306 | YW2043 *nej1*::*kanMX4* |
| YW2307 | YW2044 *nej1*::*kanMX4* |
| YW2083 | *MATa* *ade2*-M7 *his3*∆200 *leu*2- *lys*2-801 *trp1*∆63 *ura*3-52 |
| YW1993 | *MAT*a-inc::AmpR-35S *can1∆*::*GAL1*-QPCR *DNL4*-13Myc::*hisMX6* *GAL1*prm*-*HOcs *gal1*::HO *his3*∆1 *leu2*∆0 *met15*∆0 *ura3*∆0 |
| YW2033 | YW1993 *dnl4*-K282R-13Myc::*hisMX6* |
| YW2034 | YW1993 *dnl4*-D284A-13Myc::*hisMX6* |
| YW2035 | YW1993 *dnl4*-K466A-13Myc::*hisMX6* |
| YW2121 | YW1993 *lif1*∆::*kanMX4* |
| YW2162 | *MAT*a-inc::AmpR-35S *can1∆*::*GAL1*-QPCR *CDC9*-13Myc::*hisMX6* *GAL1*prm*-*HOcs *gal1*::HO *his3*∆1 *leu2*∆0 *met15*∆0 *ura3*∆0 |
| YW2163 | YW2162 *dnl4*∆::*kanMX4* |
| YW2164 | YW2162 *dnl4*-K466A |
| YW1858 | *MAT*a-inc::*LEU2 can1∆*::*ILV1*-QPCR *gal1*::HO *his3*∆1 *ILV1*prm::HOcs *leu2*∆0 *met15*∆0 *ura3*∆0 |
| YW2107 | YW1858 *dnl4*∆::*kanMX4* |
| YW2100 | YW1858 *dnl4*-K282R |
| YW2101 | YW1858 *dnl4*-D284A |
| YW2102 | YW1858 *dnl4*-K466A |
| YW2188 | *MAT*a-inc::*LEU2 can1∆*::*ILV1-*ctrl *gal1*::HO *his3*∆1 *ILV1*prm::HOcs *leu2*∆0 *met15*∆0 *ura3*∆0 |
| YW2212 | YW2188 *dnl4*-K466A |
| YW2213 | YW2188 *dnl4*∆::*kanMX4* |
| YW126 | *MAT*a *leu2*∆ *trp*1∆ *ura*3-52∆ *prb*1∆ *pep*4∆ *prc*1∆ |
| YW2189 | YW126 *dnl4*∆::*kanMX4* |
| YW2166 | *MAT*a-inc::AmpR-35S *can1∆*::*GAL1*-QPCR *LIF1*-13Myc::*hisMX6* *GAL1*prm*-*HOcs *gal1*::HO *his3*∆1 *leu2*∆0 *met15*∆0 *ura3*∆0 |
| YW2167 | YW2166 *dnl4*∆::*kanMX4* |
| YW2308 | YW2166 *dnl4*-K466A |
| YW1750 | *MAT*a-inc::AmpR-35S *can1∆*::*GAL1*-QPCR *KU80*-13Myc::*hisMX6* *GAL1*prm*-*HOcs *gal1*::HO *his3*∆1 *leu2*∆0 *met15*∆0 *ura3*∆0 |
| YW1833 | YW1750 *dnl4*∆::*kanMX4* |
| YW2309 | YW1750 *dnl4*-K466A |

**Table S1. Genotype of yeast strains used in this study.**
